# Phage Receptor-Binding Proteins for Targeted Silver Nanoparticle Delivery: Design and Evaluation

**DOI:** 10.3390/v18101145

**Published:** 2026-10-15

**Authors:** Qing He, Xiaobo Yang, Zhigang Qiu

**Affiliations:** Military Medical Sciences Academy, Academy of Military Sciences, Tianjin 300050, China

**Keywords:** receptor-binding proteins, silver nanoparticles, targeted delivery, antimicrobial resistance, biofilm-associated infections

## Abstract

Antimicrobial resistance has renewed interest in silver nanoparticles (AgNPs), but limited pathogen selectivity complicates their therapeutic use. Bacteriophage receptor-binding proteins (RBPs) recognize bacterial surface receptors and offer candidate targeting modules for AgNP delivery. However, evidence from intact-phage composites and non-silver carriers cannot establish the performance of isolated RBP–AgNP systems. In this review, we link RBP architecture and receptor recognition to ligand selection, orientation and multivalent display on AgNPs. We outline workflows for RBP discovery and receptor assignment, review conjugation strategies, and examine how Ag^+^ release and oxidative stress contribute to antibacterial activity. We propose a dual-endpoint framework to quantify nanoparticle enrichment near target bacteria and test for bactericidal gain using receptor-level controls, matched total silver doses and measurements of silver release. We discuss protein-corona masking, receptor variation and activity retention in complex infection settings, including biofilms, alongside manufacturing reproducibility. Translation further requires formulation-specific assessment of in vivo exposure, host tolerance, environmental fate and ecological risk. This framework defines the evidence needed to determine whether RBP-guided delivery improves antibacterial efficacy and safety.

## 1. Introduction

The global burden of antimicrobial resistance continues to escalate, with declining efficacy of conventional antibiotics and a marked lag in new-drug development, driving anti-infective strategies increasingly toward targeted delivery and localized, high-efficiency eradication [1,2]. In particular, the high clinical prevalence and multidrug resistance of high-risk pathogens such as the ESKAPE pathogens (*Enterococcus faecium*, *Staphylococcus aureus*, *Klebsiella pneumoniae*, *Acinetobacter baumannii*, *Pseudomonas aeruginosa*, and *Enterobacter* spp.) have made antibacterial approaches based on non-antibiotic mechanisms and amenable to engineering and modular combination a focus of antibacterial research [3,4]. Bacteriophages offer intrinsic host specificity and potent lytic activity; however, compared with deploying intact phages, phage-derived functional modules are generally more compatible with standardization and engineering [5,6]. Among these, receptor-binding proteins (RBPs) form the key molecular interface that initiates adsorption and inherently behave as “targeting ligands,” providing a molecular foundation for controllable precision antibacterial delivery [7,8].

In parallel, antibacterial nanomaterials, exemplified by silver nanoparticles (AgNPs), have evolved as potent killing platforms through multi-route mechanisms including Ag^+^ release, oxidative stress, and membrane disruption [9,10]. However, their intrinsic lack of pathogen selectivity remains a major limitation for clinical translation, as increasing antimicrobial potency is often accompanied by higher off-target exposure and safety concerns [11,12]. Conversely, decades of phage biology research have progressively revealed that RBPs function as modular bacterial-recognition elements whose specificity can be genetically and structurally engineered [7,8]. These two lines of research motivate a common design principle: separating the functions of “recognition” and “killing” into modular components that can be optimized independently.

The historical progression toward this integrated strategy is summarized in Figure 1, which highlights how advances in phage receptor recognition, protein engineering, and nanomaterial antibacterial technology have collectively created the foundation for RBP-guided antibacterial nanosystems.

Previous reviews have examined phage targeting mechanisms and the broader use of nanomaterials against resistant infections [7,13]. Here, we focus on coupling RBP recognition with silver nanoparticle delivery. Our purpose is to connect RBP architecture and receptor accessibility to construction choices and to the targeting and killing endpoints needed to evaluate an RBP–AgNP formulation. Related antibiotic-carrier and whole-phage studies provide useful evidence [14,15], but do not by themselves validate isolated RBP–AgNP constructs. Protein-corona masking [16,17,18], receptor variation [19,20,21], and silver exposure and environmental release [11,12,22,23,24] remain constraints on this proposed approach. The review therefore presents a design and evaluation framework grounded in published studies and intended for direct experimental testing.

**Figure 1:**
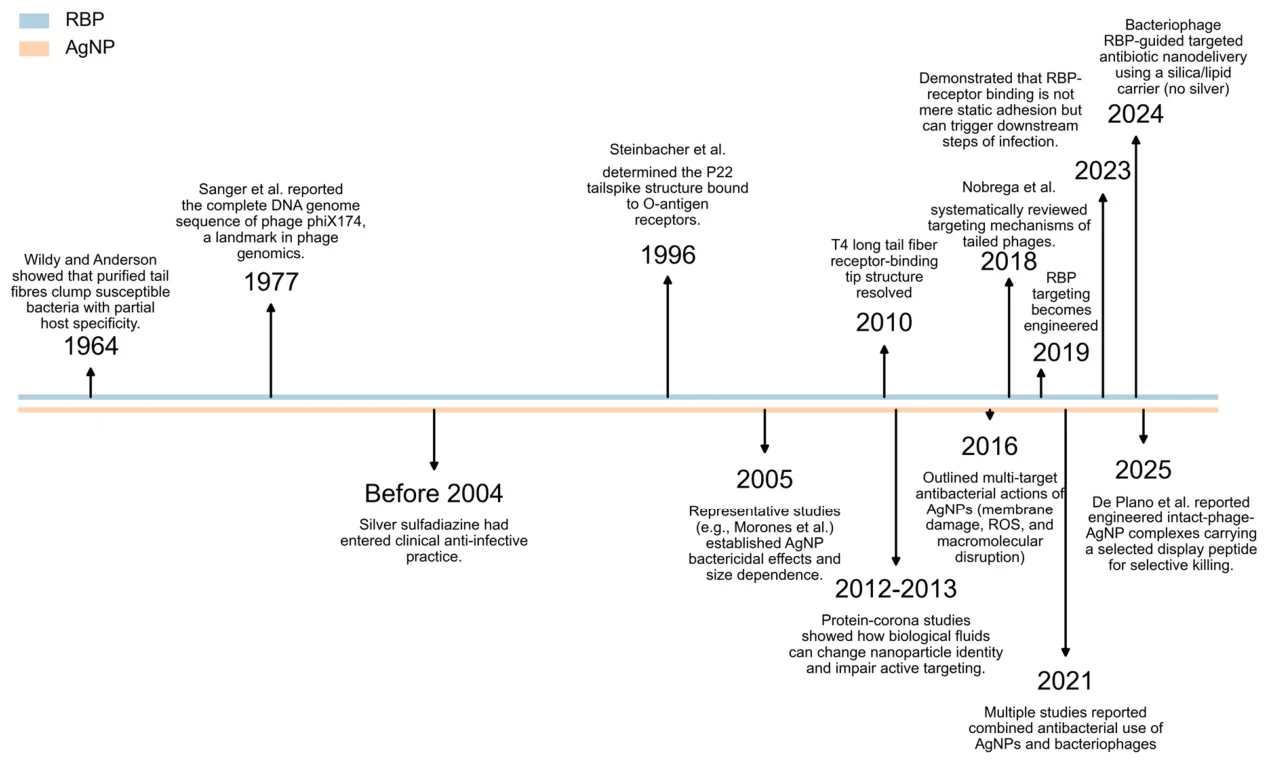
Parallel technological pathways converging on receptor-binding protein (RBP)-guided silver nanoparticle (AgNP) design. The upper blue track progresses from phage genomics and receptor recognition to engineered RBP targeting and cargo delivery; the lower orange track progresses from antimicrobial silver through AgNP antibacterial mechanisms and biological-interface constraints to phage–AgNP integration. Their convergence provides the rationale for a modular RBP–AgNP design that couples RBP-mediated recognition with AgNP antibacterial activity. Milestones are chronological; spacing is schematic [7,9,14,15,16,17,18,25,26,27,28,29,30,31,32,33,34]. Abbreviations: DNA, deoxyribonucleic acid; ROS, reactive oxygen species.

## 2. Phage RBPs: From the “First Step of Adsorption” to Programmable Targeting Modules

RBPs are key phage components responsible for adsorption through recognition of bacterial surface receptors. Owing to their modular organization and diverse receptor specificities, RBPs determine host recognition in natural phage infection and provide a structural and functional basis for engineering programmable targeting modules [7,35]. Although the term receptor-binding protein (RBP) is widely used, its operational scope can be ambiguous across phage systems and publications. In many tailed phages, the adsorption-associated tail fiber/tailspike proteins are modular, and “RBP” may refer to either the entire modular polypeptide or more narrowly to the receptor-binding domain (RBD) within it. A recurring architectural principle is that an N-terminal anchor/assembly region mediates virion attachment and tends to be relatively conserved, whereas the distal C-terminal tip/RBD is more diversified and frequently carries the dominant receptor-recognition function [36,37]. Importantly, this “C-terminal RBD-centric” description does not imply that the remaining regions are irrelevant to host recognition: shaft/stalk modules can influence spatial reach, multivalent presentation, and conformational dynamics of adsorption [7,38], thereby modulating apparent affinity and host range. Therefore, throughout this review we use “RBP” as an umbrella term when discussing virion architecture and the engineering of adsorption modules, while explicitly using “RBD” when referring to the receptor-recognition interface (often located at the C terminus). In the context of antibacterial nanomaterials, these features make RBPs attractive recognition elements for targeted nano-systems. By concentrating the nanomaterial’s bactericidal effects within the immediate vicinity of the intended pathogen, such systems aim to improve clearance while reducing off-target exposure [39].

### 2.1. Modular Architecture Links RBP Structure to Function

RBPs function as modular adsorption machines: a recurring organization in several tailed-phage RBP systems comprises (i) an N-terminal assembly/anchoring domain that docks onto the tail/baseplate, (ii) a central shaft that controls spatial reach and mechanical presentation, and (iii) a distal receptor-binding tip/RBD that provides the dominant specificity. This organization is illustrated by the AF3 model of the *Vibrio* phage vB_VpP_BT-1011 RBP in Figure 2a, where the schematic segments (1–150, 151–580 and 581–741 aa) represent proposed assembly, shaft and receptor-binding regions, respectively. Where experimentally supported, such partitioning helps explain why conservation and variability are “assigned” to different physical modules. The anchor must remain compatible with virion assembly (thus often conserved), whereas the distal tip must track receptor diversity across strains (thus diversified). This makes the C-terminal region a frequent hotspot for host-range shifts and engineering. This “conserved assembly end + variable recognition end” logic has been repeatedly observed across tail fibers and tailspikes, including well-studied systems where receptor recognition is localized to the distal region while the proximal region maintains particle attachment and overall architecture [7].

Classic examples make the modularity tangible. In T4, the crystal structure of the long tail fiber’s distal gp37 fragment reveals an intertwined trimer with a putative receptor-interacting head [34]. Even more directly, modular “tip swapping” can rewire specificity: replacing the T2 gp37/gp38 module with the corresponding proteins from PP01 redirected infectivity toward *E. coli* O157:H7 while diminishing infectivity toward the original host, an intuitive demonstration that a replaceable distal module can overwrite host range without rebuilding the entire virion [42]. Modularity also occurs in tailspikes, where the β-solenoid scaffold can contribute to a recognition/catalytic region, coupling structural rigidity with adaptable receptor interaction. A landmark structural case is the P22 tailspike, whose complex with *Salmonella* O-antigen fragments identifies the groove that recognizes a precise carbohydrate epitope on LPS [28].

RBP specificity reflects how the anchoring region, shaft, tip, and local binding pocket are assembled and presented. This is why in engineering and nano-conjugation, it matters whether we tether the whole RBP module or only the distal RBD, and whether conjugation preserves distal accessibility.

The N-to-C-terminal scheme above is not universal, and sequence position does not necessarily predict spatial exposure. The experimentally resolved T7 gp17 fiber includes a C-terminal receptor-binding region, whereas the chains in the T4 gp37 tip fold back so that both termini of the crystallized fragment lie near the collar [34,38]. In P22, the carbohydrate-binding groove lies in the central β-helical region of the receptor-binding fragment [43]. T5 pb5 provides another distinction: it is a monomeric RBP that binds the outer-membrane protein FhuA [33]. These experimental examples show why an attachment site cannot be selected solely from an N- or C-terminal label. For nanoparticle coupling, the mapped binding surface, folding requirements and native assembly state should guide construct selection, followed by direct testing of binding after immobilization. An isolated domain may retain recognition without reproducing the complete phage infection machinery.

### 2.2. RBPs Employ Dynamic Recognition Mechanisms

Modular architecture frames host recognition as a multi-step, dynamic process that can progress from contact to activation. First, adsorption can act as a trigger: receptor engagement at the distal tip can propagate conformational or mechanical signals to the baseplate/tail apparatus, switching the particle from a searching state to an infection-competent state. Structural studies of baseplate/tail rearrangements during infection provide direct evidence that binding is coupled to activation of downstream penetration/injection steps [33,44].

Within the characterized long, non-contractile-tailed phages reviewed by Goulet and colleagues, the tape measure protein (TMP), distal tail protein (Dit) and tail-associated protein (Tal) form an organizational core that accommodates diverse receptor-binding and accessory components [45]. This is a framework for the corresponding siphophage systems, not a universal description of phage adsorption devices. Lactococcal phages p2 and TP901-1 illustrate the distinction between activatable and binding-ready presentation. In p2, six RBP trimers initially point towards the capsid and undergo a large cation-associated reorientation towards the host [46]. In TP901-1, the sites already face the host; the BppU scaffold links a Dit hub to eighteen RBP trimers [47]. Accessory scaffolds thus contribute to multivalency and presentation geometry. The extended T4 fiber and compact lactococcal baseplates represent contrasting designs within the broader diversity of adsorption architectures. On AgNPs, orientation, accessible ligand number and spacing require direct evaluation; native copy numbers are not prescribed nanoparticle ligand densities, and native baseplate activation does not establish an activation requirement for the isolated RBP.

Second, some RBPs (especially tailspikes) encode enzymatic “barrier breaking” as part of recognition. This is most obvious for pathogens protected by thick polysaccharide layers, O-antigen, capsules, or biofilm matrix, where the receptor is physically shielded. Tailspike-associated depolymerases can trim or depolymerize these carbohydrates, exposing deeper receptors and accelerating irreversible adsorption. The P22 tailspike is again a teaching example: its interaction with O-antigen illustrates why “recognition” in carbohydrate-rich surfaces is often inseparable from processing of the polymeric barrier [28,48].

A particularly clear mechanistic bridge to materials design comes from diffusion and biofilm physics. Dunsing and colleagues demonstrated that in a purely polysaccharide-dominated biofilm matrix, nanoparticles face strong size-selective diffusion barriers; treatment with a phage tailspike depolymerase rapidly restores freer nanoparticle diffusion. These findings support testing depolymerase treatment before nanoparticle delivery in matrices with similar diffusion barriers [49]. In infection-relevant contexts, depolymerases can be functional anti-biofilm agents themselves. For *Acinetobacter baumannii*, the φAB6 tailspike depolymerase has been reported to inhibit and degrade biofilms, suggesting that depolymerases warrant evaluation as components of RBP-guided platforms [50,51].

Third, some phages employ multi-RBP or staged recognition strategies: different fibers/spikes can mediate reversible attachment, irreversible binding, and activation in sequence, which helps explain why swapping or mutating only one module sometimes yields partial, context-dependent changes. This matters for RBP-guided nanomaterials because conjugation can perturb affinity, geometry, and the multivalency required for productive engagement, especially on structured receptors such as O-antigen and capsule.

Receptor specificity also depends on chemical identity and location, as illustrated in Figure 2d,e. In Gram-negative bacteria, LPS O-antigen chains extend from the outer membrane, capsules form an external polysaccharide layer, and proteins such as FhuA present extracellular receptor surfaces. P22 recognizes defined O-antigen structures, while modular *Klebsiella* RBPs discriminate among capsule types [19,28,33]. In Gram-positive *Listeria*, A118 and P35 recognition depends on particular sugar substitutions in wall teichoic acids: receptor-mutant and restoration experiments identified rhamnose-dependent A118 binding and the appropriate rhamnose/N-acetylglucosamine context for P35 adsorption [20]. *Bacillus* phage SPP1 instead uses reversible association with glucosylated wall teichoic acids to accelerate recognition of the membrane protein YueB [21]. Thus, a species name does not establish receptor compatibility. Changes in surface-polysaccharide chemistry, receptor abundance or masking can alter RBP binding within a species, so specificity and coverage should be evaluated using receptor-defined isolates and appropriate receptor-altered controls, including after nanoparticle coupling.

### 2.3. Material Combinations and Functional Roles of RBPs

Because RBPs are inherently programmable bacterial-surface binders, their applications can be organized by the material platform and the function assigned to the RBP module. In diagnostic and antibacterial systems, RBPs can be combined with fluorescent reporters, drug-loaded nanocarriers, enzyme-assisted nanoparticles, antibacterial inorganic nanoparticles, and functional surfaces or coatings. These combinations convert receptor recognition into signal generation, barrier removal, localized payload delivery, direct antibacterial action, bacterial capture, or selective removal. Together, they position RBPs as modular recognition elements that can be matched to different material functions.

(i) Precision recognition probes for rapid detection and typing

RBPs can serve as high-specificity affinity reagents for pathogen capture, enrichment, and serotype/strain typing. Practical probe workflows have been demonstrated using fluorescently labeled tail fibers to recognize defined STEC serotypes in complex matrices and to validate receptor dependence with LPS-pathway mutants [52]. Quantum dot-labeled RBP 55 from phage STP55 has been combined with immunomagnetic separation to detect *Salmonella* in spiked food samples [53]. These examples link RBP-mediated recognition to fluorescence readouts; in the RBP 55 assay, antibody-functionalized magnetic beads provide capture before detection [52,53].

(ii) Host-range engineering and retargetable antibacterial biologics

Because specificity is often concentrated in distal modules, RBPs enable rational host-range engineering, from directed mutagenesis of tail fibers to swapping tip modules to retarget phages toward clinically relevant receptors [42,54]. This modularity supports phage retargeting and reusable RBP ligand libraries for functionalizing drug carriers, antibacterial particles, and material surfaces [42,54].

(iii) Barrier removal as an adjuvant: antibiofilm and anti-capsule strategies

Depolymerase-bearing RBPs or tailspikes can function as enzymatic adjuvants: weaken capsules/O-antigen/biofilm matrix to (a) enhance antibiotics, (b) enable nanoparticle penetration, or (c) restore immune access [48,49,50,51]. Accordingly, enzymatic RBPs can be used as pretreatment agents or combined with antibiotic carriers and antibacterial nanoparticles to expose receptors, open diffusion paths, and improve access to the bacterial surface [48,49,50,51].

(iv) RBP-guided therapeutic carriers and antibacterial materials

RBPs can be repurposed as targeting ligands to localize therapeutics to pathogens or infection niches. A recent proof-of-concept showed bacteriophage proteins guiding antibiotic nanodelivery, enabling selective accumulation and improved dose efficiency compared with free antibiotics [15]. This example illustrates a broader materials principle: the material provides the therapeutic payload or antibacterial effector, and the RBP provides receptor-directed localization [15]. Targeting can therefore improve local delivery efficiency when material accumulation at the pathogen reduces unnecessary systemic exposure.

(v) Surface decontamination, capture materials, and anti-adhesion interfaces

Because RBPs bind bacteria with high avidity, they can be integrated into capture surfaces, filtration materials, or coatings for selective bacterial removal. The material provides durability and processability, and the RBP provides receptor selectivity for capture, concentration, filtration, and decontamination, or for constructing anti-adhesion interfaces.

Among these material platforms, antibacterial inorganic nanoparticles provide a direct route from receptor recognition to bacterial killing. AgNPs are especially suitable because they combine multiroute antibacterial activity, including Ag^+^ release, oxidative stress, and membrane perturbation, with surfaces that support ligand functionalization [9,39,55]. Their limited intrinsic bacterial selectivity creates a clear role for RBP-mediated recognition. The following section therefore focuses on the rationale, construction, and evaluation of RBP–AgNP systems.

## 3. Quantifiable Priorities for Coupling RBPs with AgNPs

### 3.1. Rationale for Coupling RBPs with AgNPs

AgNPs provide broad-spectrum antibacterial activity but lack intrinsic bacterial selectivity. By contrast, phage RBPs act as programmable bacterial-surface ligands with receptor-level specificity, yet their value for therapy depends on whether recognition can be converted into measurable delivery selectivity rather than qualitative “binding.” Coupling RBPs to AgNPs is therefore motivated by a dose-efficiency logic: RBPs are expected to enrich AgNPs in the immediate vicinity of the intended pathogen (or biofilm niches), thereby increasing local effective exposure without increasing total silver burden. Two primary endpoints should be evaluated: receptor-dependent targeting enrichment (e.g., enrichment factor or bound fraction on target versus non-target bacteria under serum/exudate challenge) and killing gain at matched total silver dose. The latter requires comparison with free AgNPs and non-targeted controls. Dose-sparing can then be assessed at matched efficacy; a wider efficacy–safety window requires separate evidence on tissue exposure and host tolerance. These priorities establish a direct bridge from “recognition” to translational value and also define what construction strategies must preserve: RBP accessibility and receptor dependence, controllable Ag^+^ release kinetics, and colloidal stability in complex biological environments.

Complementing RBP-mediated targeting, antibacterial nanomaterials such as silver nanoparticles (AgNPs) primarily exert multi-route damage through Ag^+^ release, reactive oxygen species (ROS)/oxidative stress, membrane perturbation, and metabolic interference [9,39]. These properties have repeatedly been shown to be promising for clearing drug-resistant bacteria and biofilms [55]. However, major translational barriers remain: limited specificity dilutes effective exposure across non-target surfaces; protein-corona formation in biological fluids can remodel the nano–bio interface, promote aggregation, and attenuate activity [16,17]; and dose-dependent toxicity risks can be amplified as systemic exposure increases. In a related delivery study, Zhao and colleagues used phage-derived targeting proteins to “navigate” antibiotic nanodelivery systems, enabling selective accumulation at infection sites and drug delivery that outperformed free antibiotics, which often required ~16–32-fold higher doses to achieve comparable suppression [15]. These antibiotic-delivery results illustrate a dose advantage of targeting in the tested models [15]. These comparisons concern antibiotic delivery: the P545 RBP and SA97 endolysin cell-wall-binding domain are distinct targeting modules, and the carriers contain no silver. Their dose advantages cannot be assumed for RBP–AgNP formulations [15].

### 3.2. Experimental Workflow for RBP Identification, Construction, and Validation

For application-oriented nanomedicine, RBPs must be identified reliably and their cognate receptors established causally [7,8]. Figure 3 combines complementary early assessments without prescribing a fixed sequence. Electron microscopy provides preliminary phage morphology and structural clues, whereas genome annotation and computational screening nominate RBP candidates. Classifiers can screen tail-associated proteins [56]; HMM/Pfam annotation [57], protein language models [58] and structure prediction [37,40] can refine candidate selection and guide engineering or conjugation-site choices. Prioritized candidates then proceed through PCR amplification, recombinant expression and purification. Specific binding is tested first. Receptor causality is then examined using competition, receptor deletion/mutation or chemical/enzymatic disruption, ideally with complementation-based recovery [52].

### 3.3. Construction Strategies, Evaluation Criteria and Translational Considerations for RBP–AgNPs

We outline three candidate approaches for RBP–AgNP construction: noncovalent self-assembly, affinity/coordination immobilization, and covalent conjugation. Selection should be guided by three testable criteria: whether targeting is retained, whether Ag^+^ release remains controllable, and whether the construct remains stable under complex conditions [16,17].

Evaluation should therefore establish receptor-dependent targeting enrichment and test for killing gain relative to free AgNPs and non-targeted controls at matched total silver dose. Particle dose and Ag^+^ release should also be reported, alongside the host–cell viability assessment illustrated in Figure 4. Key physicochemical parameters should be reported in parallel, including particle size and polydispersity index (PDI), ζ-potential, silver loading, Ag^+^ release profiles, salt/serum stability, and storage stability [59]. For example, De Plano and colleagues constructed AgNP@Li5 complexes by combining AgNPs with engineered Li5 phage, achieving highly selective inhibition against Li5-recognized *Escherichia coli* strains with minimal impact on non-recognized bacteria [14]. AgNP@Li5 contains an intact M13-derived phage displaying an exogenous targeting peptide, rather than an isolated RBP. Its reported selectivity is evidence from the tested bacterial strains and does not establish in vivo efficacy or host safety for isolated RBP–AgNP constructs [14].

At the application level, potential applications for evaluation include biofilm-associated infections, chronic infections/persister populations, and localized delivery formats (e.g., dressings, gels, coatings, or aerosols) [61,62]. Ultimately, translational feasibility will depend on whether the RBP binding interface remains accessible after conjugation and can maintain targeting in protein-corona environments [16,18], whether robustness against receptor variation and escape can be achieved (e.g., via multivalent display or multi-RBP combinations) [7], and whether standardized manufacturing and comparable evaluation frameworks are established [59].

## 4. Challenges and Translational Barriers

### 4.1. Protein Corona Formation and Masking in Biological Fluids

In complex media such as serum and wound exudates, nanoparticles typically acquire a dynamic protein corona within minutes, which effectively rewrites the interfacial charge and chemical composition [16,63]. As a consequence, the receptor-binding surface of a receptor-binding protein (RBP) displayed on the nanoparticle can be partially masked or forced into an unfavorable orientation, and even local conformational flexibility may shift, ultimately flattening the originally clear binding contrast between target and non-target bacteria; in severe cases, the difference can shrink to near background [17,63]. Targeting observed in phosphate-buffered saline (PBS) may not be retained in biological fluids, contributing to an in vitro–in vivo mismatch. Retention of targeting after corona formation should therefore be established and reported as a core line of evidence for RBP-guided systems [64]. In the nanodelivery field, this point has been demonstrated experimentally: Mirshafiee and colleagues showed that the protein corona can screen the active sites of targeting ligands, leading to a marked reduction in active-targeting performance, highlighting corona-driven loss of ligand accessibility even when ligand density remains high [17].

Engineering a beneficial corona remains a proposed strategy for RBP–AgNPs, and retention of RBP targeting must be demonstrated directly. Oh and co-workers leveraged supramolecular interactions to construct a “protein-corona shield” that modulates nanoparticle–biosystem interactions, but this tumor-targeting nanocarrier study does not demonstrate preservation of an RBP binding interface on AgNPs [64]. Competitive adsorption may mask that interface or alter its orientation; binding and killing should therefore be assessed after corona formation in the relevant biological medium [17,18].

### 4.2. Post-Conjugation Activity Retention and Long-Term Stability

After RBP immobilization on a nanoparticle surface, retained functional activity becomes the primary quality attribute [16,63]. On the one hand, the recognition interface can be pushed into an unfavorable, receptor-inaccessible orientation by steric crowding; on the other hand, surface interactions may subtly distort the protein conformation, thereby reducing apparent affinity [17,63]. Meanwhile, under more realistic conditions, such as high ionic strength or intense competition from abundant proteins, the formulation can readily undergo aggregation, desorption, or ligand displacement [18], and during storage it may gradually lose performance due to freeze–thaw cycles, changes in buffer composition, or shear stress. Conjugation density and loading should therefore be interpreted together with functional measurements [17,59]. A more defensible assessment should, at minimum, address three points: (i) receptor-dependent binding specificity across target and non-target bacterial species [17,59]; (ii) whether the construct resists displacement and competitive adsorption in serum or high-salt environments [18,63]; and (iii) whether particle size, ζ-potential, binding performance, and the killing gain remain reproducible after a defined storage period [59].

Protein-corona composition is dynamic. As emphasized by Singh and colleagues in discussions of the in vivo protein corona, corona composition can evolve with time and physiological context, thereby shaping circulation, clearance, and the apparent targeting behavior of nanoparticles [65].

### 4.3. Receptor Variation/Escape and Host-Range Drift

Receptor-dependent targeting can appear exquisitely precise, but it carries a practical liability: single-point failure [7]. Clinical isolates often differ far more than laboratory reference strains, and receptor-level structures, such as LPS O-antigen, capsular polysaccharides, and outer-membrane proteins can vary rapidly across strains [66]. Bacteria can further diminish RBP binding through receptor modification, receptor loss, or even downregulation of receptor expression [8,66]. Consequently, targeting based on a single RBP is prone to escape, and the apparent host spectrum can drift substantially across isolate panels [8]. From an application standpoint, the key is to find an engineerable balance between high specificity and robust coverage. One approach is to increase effective binding probability through multivalent display; another is to combine multiple RBPs and/or multi-receptor targeting within the same construct, borrowing the “cocktail” concept to broaden coverage, and to quantify performance across panels of clinical isolates [7,66].

Whole-phage studies provide a rationale for investigating this approach. In modeling work, Marchi and colleagues reported that combination therapies composed of phages targeting independent receptors can improve treatment outcomes while reducing the probability that pathogens simultaneously evolve resistance to all components of the combination [67]. These models motivate testing multi-receptor RBP display, but do not establish its ability to prevent escape in RBP–AgNP systems [67]. More application-oriented evidence comes from Rotman and co-workers, who rapidly designed a phage cocktail from a well-characterized phage library and selectively reduced the burden of carbapenem-resistant *Klebsiella pneumoniae* in the murine gut niche, while also driving the loss of key virulence factors that functioned as phage receptors [68]. Together, these findings support quantification of coverage, escape costs, and robustness in realistic isolate panels [67,68].

### 4.4. In Vivo Fate and the Safety Window

The antibacterial efficacy of silver nanoparticles (AgNPs) depends largely on Ag^+^ release and interfacial reactions [4,39]. However, once systemic exposure increases, cytotoxicity, inflammatory responses, and the potential risk of silver accumulation often rise in parallel, so that efficacy and toxicity can appear to be “turned up” together [39,60]. Compounding this challenge, in vivo distribution and clearance of nanoparticles are strongly governed by particle size, surface chemistry, and protein-corona formation [69,70]. As a result, nanoparticles frequently accumulate in the reticuloendothelial system (RES), creating a clinically relevant paradox in which the target site may receive an insufficient effective dose while off-target tissues experience excessive exposure [69]. Accordingly, translational assessment should use clinically meaningful endpoints: under matched efficacy, can the required silver dose be reduced, can the tissue exposure profile be improved, and is the efficacy–safety window genuinely widened [59]? The most compelling translational argument for RBP-guided targeting is a measurable shift in the efficacy–safety balance toward a more usable regime [59]. Clinical implementation also requires timely identification of strains bearing accessible receptors. Local and systemic formulations need different exposure and safety assessments; efficacy in one setting cannot establish suitability for the other. Clinical-isolate coverage, targeting in protein-rich infection environments, tissue silver exposure and host tolerance should therefore be evaluated for the intended indication and administration route [7,69,70,71,72].

Once the efficacy–safety window has been quantified, development also depends on manufacturability and reproducibility, including consistent batch composition and functional performance [71]. Without this, even strong in vivo results are unlikely to progress through development and regulatory evaluation [71].

### 4.5. Scalable Manufacturing and Quality Control

As a platform moves from proof-of-concept toward translation, process control and product quality become major development priorities [72,73]. RBPs are biological macromolecules and therefore carry inherent risks of batch-to-batch variability and degradation [73]. AgNPs, in turn, can vary in particle-size distribution and dissolution (release) kinetics [59,73]. After coupling, additional sources of variability are introduced, including conjugation density, orientation consistency, and differential responses to protein-corona formation [72]. Thus, without a quality framework that is auditable and reproducible, even a highly effective system is unlikely to mature into a product [73].

From a translational and regulatory perspective, a more practical route is to define critical quality attributes (CQAs) up front and then operationalize robust release specifications [73,74]. At a minimum, these should include particle size and polydispersity index (PDI), ζ-potential, silver loading, Ag^+^ release profiles, conjugation density and activity retention, serum stability and storage stability, endotoxin and residual reagents, as well as acceptable ranges for inter-batch consistency [59,75]. Equally important is to connect “characterization–function–safety” into a coherent evidentiary chain, namely, that changes detected in physicochemical attributes can explain shifts in biological performance, and that performance changes can in turn be mapped onto the efficacy–safety window [59]. Establishing this linkage is what distinguishes a system that can be advanced through translational development from one that remains a visually compelling concept demonstration [74].

Phage medicinal-product standards concern preparations containing phages and do not constitute a product-specific standard for purified RBP–AgNPs. In particular, European Pharmacopeia chapter 5.31 addresses phage therapy medicinal products [76]. These standards provide regulatory context, but their direct applicability to an RBP–nanoparticle product must be assessed separately; they are not evidence of its translational readiness.

### 4.6. Environmental Fate and Ecological Risk

Environmental assessment should extend from production and clinical use to disposal. Silver released from formulations or discarded dressings can enter wastewater and subsequently reach effluent or biosolids. A pilot wastewater-treatment study found that silver nanoparticles associated with solids and were largely transformed to silver sulfide [22]. Sulfidation can reduce dissolved-silver availability and toxicity, but transfers and transformations do not eliminate the total silver burden [23]. In a terrestrial mesocosm study, AgNP-containing biosolids affected plant and microbial responses under the tested exposure conditions [24]. Such findings preclude inferring ecological safety solely from efficient bacterial targeting.

Silver-resistance determinants, including systems associated with silver binding and efflux, have been identified in bacteria [77]. This does not establish that a particular RBP–AgNP formulation will select resistance, but supports including resistance selection in lifecycle assessment. Development should therefore measure silver release during use and disposal, characterize its transformed species, and evaluate effects on aquatic organisms and wastewater or soil microbial communities at relevant exposures. Claims that targeting reduces ecological risk require a measured reduction in released silver together with appropriate ecotoxicity evidence; a lower antibacterial dose alone is insufficient.

## 5. Outlook

Looking ahead, for RBP–AgNP systems to move from “conceptually attractive” demonstrations to platforms that are genuinely usable and translatable, progress is needed in four areas: targeting modules, evaluation frameworks, system design, and manufacturing pathways.

First, a scalable, data-driven “RBP–receptor” predictive and targeting-module library should be built on more systematic evidence accumulation [66]. On the one hand, the RBP identification loop should be continuously strengthened by depositing standardized, high-quality pairings that link RBP sequence/structure → receptor type → covered strains/serotypes → binding kinetics. On the other hand, these curated pairwise datasets should be fed back into machine-learning models and structure-prediction pipelines to improve host-range and receptor-use predictions. The practical goal is a “plug-and-play targeting platform”: when faced with a specific clinical pathogen or serotype, an appropriate RBP module could be selected and assembled in a standardized manner onto a predefined nanoscale bactericidal scaffold [56,66].

Second, evaluation and modeling should be aligned more closely with real infection contexts. To reduce in vitro–in vivo discrepancies, future work will require clinically relevant infection models and standardized evaluation workflows, for example, verifying targeting retention under serum/exudate-containing conditions; assessing enrichment and clearance in mature biofilms, mucoid phenotypes, polymicrobial communities, or chronic-infection models; and incorporating into a minimum reporting checklist metrics such as kill gain under matched dose, enrichment factor, corona effects, stability, and batch-to-batch consistency. Only when studies are comparable within the same control and endpoint framework can the true incremental value of RBP-mediated targeting be recognized and iterated efficiently.

Third, system design is likely to evolve from “targeting + killing” toward more integrated, multifunctional and stimulus-responsive architectures [78]. For RBP–AgNPs, conjugation alone does not establish retained targeting, resistance to receptor escape, or a wider safety window. A more promising direction is to co-design targeting, controlled release, and escape resistance within a single construct: using interfacial engineering to reduce nonspecific adsorption while preserving RBP accessibility [64]; employing microenvironment-responsive mechanisms (e.g., pH-, enzyme-, or ROS-responsive elements) to tune Ag^+^ release kinetics, reduce peak exposure [78,79], and raise effective dosing at the infection site; and adopting multivalent display, multi-RBP combinations, or replaceable targeting modules to mitigate single-point failure and maintain robustness under strain diversity and receptor variation pressure.

Finally, a clear translational roadmap is needed to move from laboratory studies to GMP-compatible production and ultimately clinical testing. At the candidate-selection stage, critical quality attributes (CQAs) and release specifications should be established (e.g., size/PDI, ζ-potential, silver loading, Ag^+^ release profiles, conjugation density and activity retention, serum stability, storage stability, endotoxin and residuals) [59]. At the validation stage, we propose using standardized animal models to assess efficacy and safety together, including whether the required silver dose can be reduced at matched efficacy. At the process stage, manufacturing and quality control must be scalable and reproducible [59]. Ultimately, preclinical and clinical studies should align delivery routes and endpoints with appropriate indications (e.g., localized infections and biofilm-associated infections).

## 6. Conclusions

In summary, RBP–nanomaterial antibacterial composites are designed to translate the “host specificity” of bacteriophages into an engineerable and deployable form of delivery selectivity, and couple this selectivity with multi-route killing modules such as silver nanoparticles (AgNPs) in a complementary manner. On the one hand, RBPs enable receptor-directed recognition and enrichment on the target bacterial surface, pulling effective exposure into the immediate pericellular microenvironment; on the other hand, AgNPs deliver localized bactericidal effects through Ag^+^ release, oxidative stress, and membrane perturbation. The aim is therefore to improve delivery selectivity and test whether the silver dose can be reduced at matched efficacy. Any improvement in the efficacy–safety window requires separate evidence on tissue exposure and host tolerance.

Practical use requires a reproducible and auditable evidence chain. Three requirements are central. First, receptor-level controls must establish the RBP–receptor relationship. Second, after conjugation, RBP orientation and activity must be retained under conditions that better approximate real biological environments, such as serum, high ionic strength, or strong competitive adsorption, so that corona masking, desorption/displacement, or aggregation does not gradually distort or erase targeting. Third, under strictly matched total silver dose, with particle dose and Ag^+^ release also reported, targeting should be demonstrated to provide a quantifiable gain in bactericidal activity. At the same time, key physicochemical and product attributes, including particle size and polydispersity index (PDI), ζ-potential, silver loading, and Ag^+^ release profiles, should be reported to ensure that the relationship between characterization and function is reproducible and readily interpretable.

Future work should prioritize adaptable RBP libraries, multivalent or multi-RBP strategies to address receptor escape, and standardized manufacturing alongside in vivo safety evaluation. For isolated RBP–AgNP systems, these advantages remain to be established through direct, formulation-specific evaluation that also includes environmental fate and ecological risk.

## Figures and Tables

**Figure 2:**
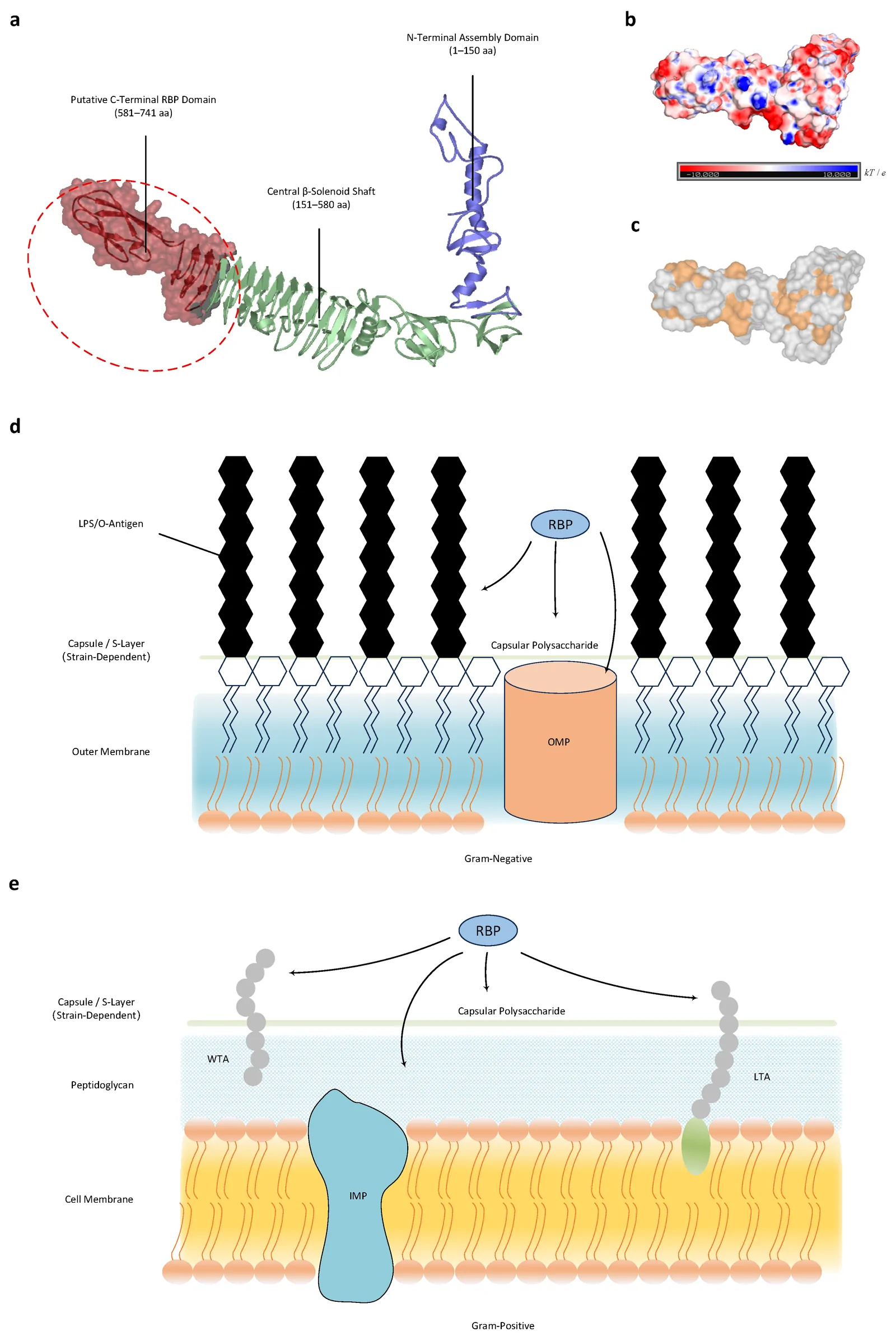
Modular architecture of RBPs, head-domain characteristics, and receptor distribution. (**a**), Modular architecture of an RBP. The RBP shown (derived from *Vibrio* phage vB_VpP_BT-1011) was predicted using AlphaFold3 (AF3 [40]) and visualized using PyMOLversion 3.1.1 [41] for illustrative purposes only. Proposed regions comprise an N-terminal assembly/anchoring domain, an intervening linker/connector, and a C-terminal receptor-binding domain (RBD); the termini are indicated. (**b**), Surface electrostatic potential distribution of the putative head domain of the receptor-binding protein (RBP) (scale: −10 to 10 *k*T/*e*). (**c**), Surface hydrophobicity distribution of the putative C-terminal receptor-binding domain (RBD). Hydrophobic patches are highlighted in orange. (**d**), Schematic surface features of Gram-negative bacteria, including LPS/O-antigen, outer-membrane proteins (OMPs), and capsular polysaccharides/S-layer, which can serve as RBP receptors. (**e**), Schematic surface features of Gram-positive bacteria, including wall teichoic acid (WTA), lipoteichoic acid (LTA), and capsular polysaccharides/S-layer. IMP denotes an integral membrane protein. The relevant receptor must be accessible on the surface of the target bacterium; specialized contexts such as biofilm-associated receptors are not explicitly depicted. The domain annotations are illustrative and require experimental validation.

**Figure 3:**
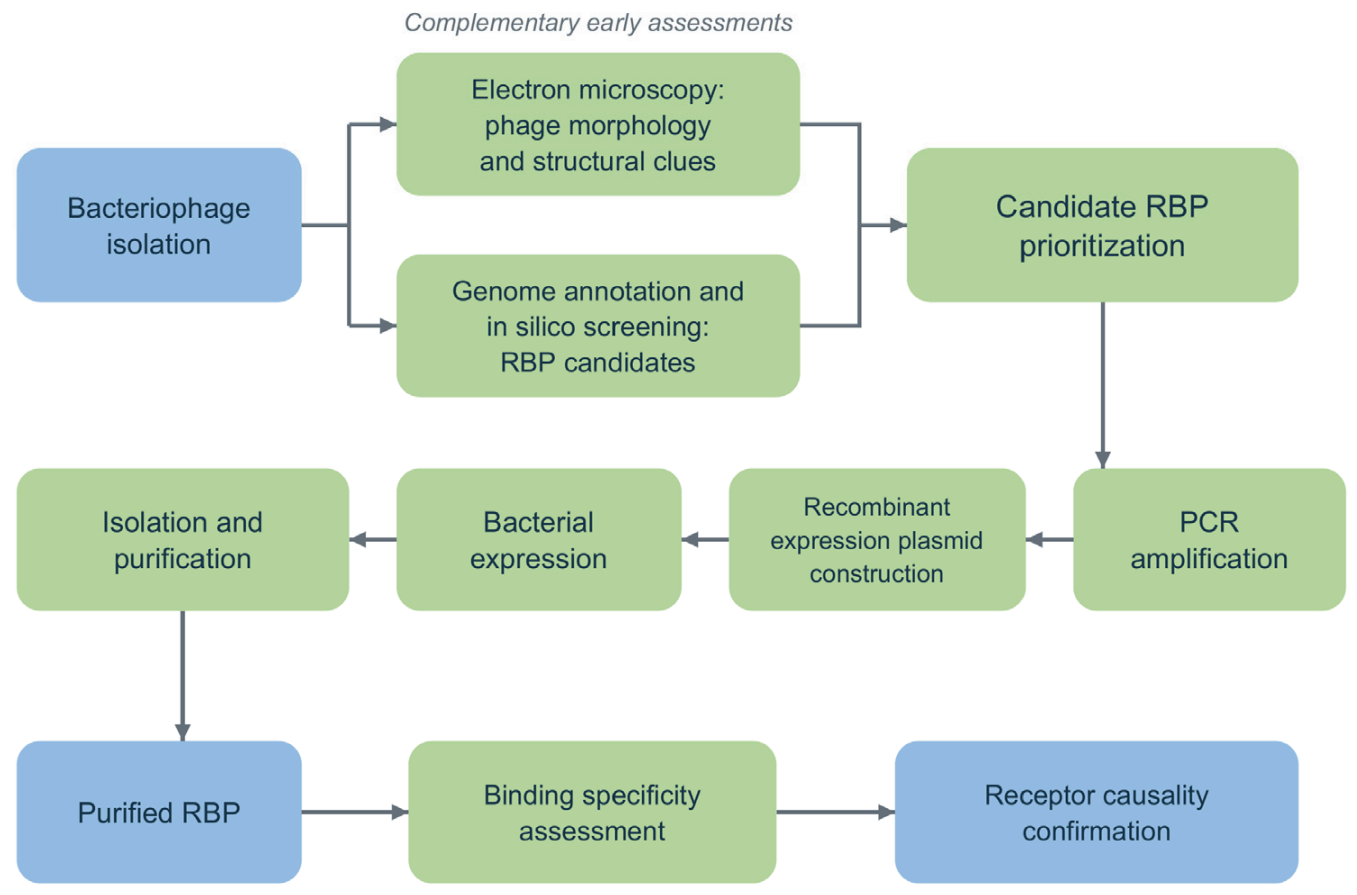
Workflow for RBP prediction, screening, and identification. Candidate RBPs are nominated through computational triage and screening, alongside preliminary phage morphology characterization, to yield relatively purified RBP preparations, which are then subjected to stepwise experimental validation in binding and receptor-confirmation assays.

**Figure 4:**
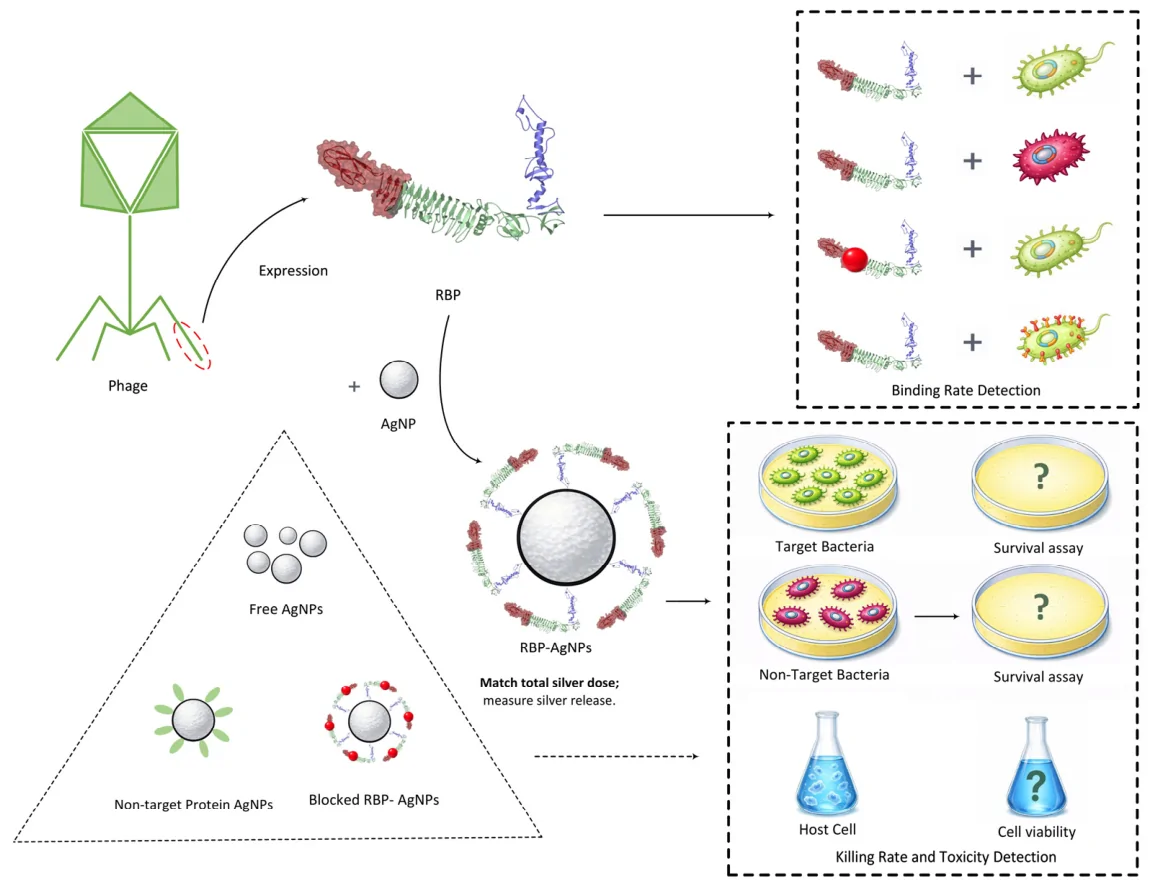
Two-dimensional evaluation framework integrating Targeting × Killing. Tier 1 assesses receptor-dependent binding (upper-right) using RBP + target bacterium, RBP + non-target bacterium, competitive inhibition, and receptor modification/disruption. Tier 2 evaluates bacterial survival and host–cell viability for RBP–AgNPs (lower-right), with three comparator/control groups shown lower-left. Question marks identify the selective-killing and host-toxicity outcomes to be measured. Compare formulations at matched administered total silver dose, or initial silver concentration and exposure duration in vitro, and quantify silver release. The scheme and molecular images depict the evaluation concept [4,15,60].

## Data Availability

No new data were created or analyzed in this study. No new sequence data were generated in this study. The amino acid sequence of VBVPP, annotated as a tail fiber protein from *Vibrio* phage vB_VpP_BT-1011, was retrieved from the NCBI Protein database under accession number YP_010649823.1 (https://www.ncbi.nlm.nih.gov/protein/YP_010649823.1, accessed on 12 October 2026).
